# Influence of Basic Health Care Provision Fund in improving primary Health Care in Kano state, a descriptive cross-sectional study

**DOI:** 10.1186/s12913-023-09708-w

**Published:** 2023-08-22

**Authors:** Zainab Auwalu Ibrahim, Kennedy Diema Konlan, Yoon Moonsoo, Paul Kwetishe, Jongsoo Ryu, Da Sol Ro, So Yoon Kim

**Affiliations:** 1 Federal Ministry of Health Dept. of Hospital services Abuja, Abuja, Nigeria; 2 Department of Public Health Nursing, School of Nursing and Midwifery, University of Health and Allied Sciences, Ho, Ghana; 3 Institute of Tropical Medicine, College of Medicine, Yonsei University, Seoul, 03722 Korea; 4 Division of Medical Law and Bioethics, Department of Medical Humanities and Social Sciences, Yonsei University College of Medicine, Seoul, Korea; 5 Department of Medical Humanities and Social Sciences College of Medicine, Yonsei University, Seoul, 03722 Korea

**Keywords:** Community Health Fund, Basic Health Care Provision Funds, Basic Minimum Package of Health Services, Primary Health Care Facilities

## Abstract

**Background:**

The Basic Health Care Provision Fund (BHCPF) is a direct financial investment that funds Primary Healthcare (PHC) to improve the quality of services. This study assessed the influence of the BHCPF in improving PHC services.

**Methods:**

A descriptive cross-sectional study was conducted among PHC workers in 100 facilities randomly selected from the 484 designated PHCs for implementing the BHCPF project in Kano state. Using multiple sampling methods, 200 healthcare workers in PHC facilities were selected and assisted by trained data collectors to respond to the questionnaires. Chi-square analysis was used to show associated factors, while binary regression analysis was used to determine the relationship between factors influencing the BHCPF implementation in PHC.

**Result:**

The findings showed healthcare workers had higher awareness (61.7%) and good utilization (57.1%) of BHCPF. Challenges of the BHCPF implementation were insufficiently skilled health professionals (85%), lack of data management capacity (52.6%), low community participation and awareness (52.0%), delay in releasing funds (60.7%), poor infrastructure (87.8%), and weak financial management and accountability system (58.2%). Healthcare professionals having a diploma were four times more likely to have the National Health Management Information System (NHMIS) in their facilities (AOR = 4.955, 95% CI = 1.120–21.036; P-value 0.035) than those without. Primary healthcare facilities were two times more likely to have the NHMIS (AOR = 2.549, 95% CI = 1.167–5.566: P-value 0. 019) than health post.

**Conclusion:**

The factors that influenced PHC facilities to promote the implementation of BHCPF included: periodic evaluation of the facilities, availability of functional storage facilities, and improving the standard of care in PHC facilities. There is a need for retraining healthcare workers and creating more community awareness of the BHCPF.

## Background

The World Health Organization indicates that countries must attain universal health coverage (UHC) to ensure the health of all citizens [1]. Primary health care (PHC) acts as the “programmatic engine” for achieving UHC, providing health security, and achieving health-related sustainable development goals (SDGs) [2]. In attaining the UHC, socioeconomic barriers and gender inequalities must be eliminated to promote the health and well-being of all citizens, and this can be addressed through PHC [3]. Primary health care is defined as a whole-of-society approach to health that prioritizes people’s needs and preferences (as individuals, families, and communities) as early as possible along the continuum of care from health promotion and disease prevention to treatment, rehabilitation, and palliative care, and as close as possible to people’s everyday environment [3]. The PHC’s goals are consistent with UHCs; it strives to ensure that all people have access to essential health services, medicines, and vaccinations that are safe, effective, and affordable [3]. Primary healthcare initiatives facilitate health systems in improving their performance by lowering overall healthcare spending while simultaneously boosting population health and access to care [4].

Following the passage of the National Health Act (NH Act) in 2014, Nigeria established the Basic Health Care Provision Fund (BHCPF), which is pivotal in the country’s efforts to enhance the health system, attain UHC, and improve health service indicators [5]. The BHCPF delivers primary and secondary healthcare services by providing the Basic Minimum Package of Health Care Services (BMPHS) and Emergency Medical Treatment - EMT [6]. Nigeria makes an annual allocation of at least 1.0% of the consolidated revenue (as recommended by WHO) and international donor partners’ funds, other sources, and contributions from the private sector to support the BHCPF [6]. Part of the BHCPF (45%) is disbursed to the National Primary Health Care Development Agency (NPHCDA) to strengthen the PHC system through the provision of essential drugs, vaccines, and consumables [5]. The funds will also provide for maintaining facilities, laboratories, equipment, transportation, and human resources development for quality PHC [5]. Basic Health Care Provision Fund seeks to minimize out-of-pocket expenditure by 30% in five years while increasing financial risk protection through health insurance [5].

The Nigerian PHC system is in disarray, with just about 20% of the country’s 30,000 PHC facilities functional [7]. Primary healthcare service delivery is abysmal, and the health indices are still among the worst globally, with promotive and preventive healthcare interventions underutilized [7]. Nigeria failed to meet the commitment of health care expenditure made in the Abuja declaration to devote at least 15% of annual expenditures to improve the health sector [8, 9]. A study carried out in Kano state assessed 49 PHC facilities. The outcome revealed considerable gaps in service delivery and a lack of adherence to the minimum requirements for PHCs (based on NPHCDA’s minimum standards for PHCs). The study also shows the shortage of healthcare professionals, notably doctors, and nurses, in about 90% of the PHC facilities, while only 21.5% of deliveries were attended by skilled birth attendants [8, 9]. The PHC facilities must have adequate infrastructure to improve service delivery efficiency, efficacy, and timeliness [8]. Although the BHCPF project has been in operation in Kano state for over three years, there has been limited information that evaluates the ability of the PHC facilities to use these funds generated by the BHCPF to provide quality health care services in local communities. This study assessed the influence of the BHCPF in improving PHC services in Kano state.

## Methods

### Design and settings

A descriptive cross-sectional study was used among healthcare workers selected from PHC within Kano state – one of the 36 states in Nigeria’s federal republic. The state is located in northern Nigeria and has 1,346 healthcare institutions (2 tertiary, 34 secondary, 1066 primary healthcare centers, and 244 private hospitals) [10]. The PHC Management Board selected 484 facilities to participate in the BHCPF intervention program. The health expenditure for 2020 was around 26,901 billion Naira (over 64 million dollars), and the spending for 2021 was approximately 30,719 billion Naira (estimated at 73 million US Dollars) [11].

### Population and Sample

The study’s population was PHC workers (Nurses, Doctors, and Community healthcare workers) from 484 facilities chosen for the BHCPF project’s enrollment. In Nigeria, not all cadre of health care professionals are found in all primary health care settings. Therefore, we focused our study on assessing all healthcare professionals who were in direct clinical contact with the patient for extended periods. However, other health professionals like laboratory technicians, radiologists, and other paramedical staff were included even though their numbers were limited. The random sampling method was adopted using a paper lottery method to select 100 PHC facilities. The G*Power 3.1.9.7 was used to calculate the sample size. Noting a test statistic of chi-square test of goodness of fit test (contingency tables) with a medium effect size (0.03), at a power = 0.95, an alpha = 0.05, and a sample of 172 was computed. Using a possible drop rate of 16% that translated to 28, a sample of 200 participants was determined. The computed sample size was 200 healthcare workers who were contacted by researcher assistants in all the selected facilities to respond to the questionnaire individually.

### Research tool

The data collection tool was a survey questionnaire uploaded and administered through google forms. The questionnaire consisted of 4 sections made of closed-ended and some open-ended questions. The first sections consist of social demographic data, then the level of knowledge on BHCPF, the capacity of PHC facilities in achieving the role of BHCPF for the provision of improved healthcare services, and the PHC’s challenges in utilizing the BHCPF funds. A pretesting of the study tool was conducted on 20 healthcare workers from non-participating PHC facilities in Kano state to assess test-retest reliability. Internal consistency reliability was assessed using Cronbach’s alpha coefficient. Using SPSS version 25.0 software, Alpha Cronbach’s score for the entire tool was 0.71. The section on knowledge was 0.73, capacity 0.92, and challenges 0.82.

### Data collection and analysis

Research assistants received two days of training on the study tool, consenting processes, and ethical considerations before data collection. Respondents received an electronic link to the Google form through their emails or any social media handle like WhatsApp or Facebook after they agreed and signed informed consent. All the study respondents were given ample time to complete the questionnaire at their own pace after submitting it to the first author. Data were checked for completeness, downloaded into an Excel spreadsheet, and then transferred to Statistical Package for SPSS version 25.0 program for analysis. Each of the discrete variables was expressed as percentages. Categorical data were analyzed through cross-tabulations using the chi-square test statistics. Variables identified as statistically significant (p-value ≤ 0.05) were entered into a binary logistic regression model to determine the predictors of BPHC.

## Result

### Sociodemographic characteristics

Most (51.5%) of the 196 PHC healthcare workers were between 30 and 45 years and females (51.5%). The majority of the participants were community health workers CHEW (83.2%) and had high school diplomas (93.9%). Most of the respondents were from health facilities that were PHC centers (65.3%), comprehensive health centers (12.8%), and health posts (21.9%). Healthcare workers had 1–10 years of working experience (79.6%). The demographic and work characteristics of the health workers are shown in Table 1.

**Table 1:** Distribution of sociodemographic characteristics

	Frequency	Percentage
Sex		
Female	101	51.5
Male	95	48.5
Profession/Occupation		
Community Health Extension worker	163	83.2
Midwives	8	4.1
Nurses	5	2.6
Others	20	10.2
Highest education attained		
Degree	12	6.1
Diploma	184	93.9
A facility that best describes where you work		
comprehensive health care	25	12.8
Health post	43	21.9
Primary health care	128	65.3
Duration working in current position/ profession		
1–10	156	79.6
11–20	36	18.4
21–30	4	2.0

### Level of knowledge on BHCPF

The study showed that PHC healthcare workers have adequate knowledge of BHCPF (61.7%), and (61.7%) understood the BHCPF concept. Most of the respondents (89.8%) had good knowledge of BHCPF based on the training they received from the healthcare authorities. Health workers (86.7%) showed that their information source was from healthcare authorities. The PHC healthcare workers (55.6%) indicated that they need additional clarification on how the 45% BHCPF will be utilized to strengthen the primary healthcare system. Healthcare providers (30.6%) want to know more about how the National Health Insurance Scheme will manage the 50% allocated funds for purchasing the BMPHS. The respondents indicated that they make quarterly returns (90.8%), and others (75.0%) showed that the money was used for its intended purpose. The related knowledge variables of respondents on BHCPF are shown in Table 2.

**Table 2:** Distribution of participants knowledge on BHCPF

	Frequency	Percentage
Have adequate knowledge of BHCPF		
No	75	38.3
Yes	121	61.7
Confidence of awareness of the concept of BHCPF		
Not too well	39	19.9
Poorly	6	3.1
Very well	52	26.5
Well	99	50.5
Knowledge acquired based on training		
No	20	10.2
Yes	176	89.8
Sources of information regarding the BHCPF		
Colleges	21	10.7
Health care authority	170	86.7
Media	4	2.0
Pamphlets	1	0.5
Aspects requiring education on BHCPF		
Ensuring the provision of BMPHS by applying 50% of the funds	60	30.6
Providing Emergency Medical Treatment, with 5% of the BHCPF	27	13.8
Strengthening the PHC system, with 45% of BHCPF	109	55.6
Frequency of making returns		
Half-yearly	15	7.7
Quarterly	178	90.8
Annually	3	1.5
Use of BHCPF funds for the intended purpose		
No	49	25.0
Yes	147	75.0
Return system included in the training received on BHCPF		
No	6	3.1
Yes	190	96.9

### The capacity of the PHC to utilize the BHCF

The healthcare workers showed the capacity of PHCs to provide adequate healthcare services, indicating inadequate staff (86.2%) to deliver BMPHS using the BHCPF. A significant number of health workers (94.4%) agreed that the BHCPF project allows periodic evaluation of the healthcare facilities, and others (85.2%) indicated it permits evaluation of the BHCPF performance in each selected PHC facility. The grading outcome of the facilities was good (48.0%), very good (34.2%), and excellent (10.2%). Only a few (47.7%) of the respondents agreed with established data management/record-keeping systems in PHC centers. Most of the respondents (73.5%) confirmed the lack of an annual quality improvement strategy to improve the skills of healthcare professionals using the BHCPF funding. Most health workers (85.7%) disclosed that they don’t have displayed information on BHCPF and BMPHS in their facilities for community awareness, and also (79.6%) confirmed inadequate health information about BHCPF to the community. A good number of the participants (66.3%) agreed on the availability of emergency care for maternal, neonatal, and child health in the PHC facilities, and (86.2%) identified poor referral systems. Healthcare workers (57.1%) highlighted that the BHCPF funds were used to improve the standard of PHC services in their facilities, and others (77.6%) confirmed insufficient funds for community outreach. In comparison, (82.1%) indicated the lack of funds for community prevention. The capacity of the PHC to utilize the BHCPF is shown in Table 3.

**Table 3:** Capacity of the PHC to utilize the BHCPF

	Frequency	Percentage
Adequate staff in facilities to provide BMPHS under the BHCPF		
No	123	62.8
Yes	73	37.2
Awareness that BHCPF allows for periodic evaluations of facilities.		
No	11	5.6
Yes	185	94.4
Evaluation of the performance of the BHCPF		
No	29	14.8
Yes	167	85.2
Established National Health Management Information System		
No	103	52.6
Yes	93	47.4
Annual quality improvement strategy implemented in the facility		
No	107	55.6
Yes	87	44.4
Functional storage facilities for health commodities		
No	55	28.1
Yes	141	71.9
Display of relevant information in the PHC facility on BHCPF and BMPHS		
No	168	85.7
Yes	28	14.3`
Adequate health information on BHCPF to the community		
No	156	79.6
Yes	40	20.4
adequate facilities for the management of emergencies		
No	66	33.7
Yes	130	66.3
Prompt referral of clients, in line with the standard operating procedures		
No	169	86.2
Yes	27	13.8
BHCPF Improving the standard of PHC facilities		
No	16	8.2
Yes	180	91.8
Sustainability of the BHCPF project		
No	155	79.1
Yes	41	20.9
BHCPF funds use to strengthen PHC services in facility and community		
No	84	42.9
Yes	112	57.1

### Factors associated with BHCPF in Kano State

There was a statistically significant relationship between PHC facilities (43%, *Χ*² =11.752, n = 196) and the lack of staff to provide BMPHS. The lack of adequate staff was among comprehensive health care (6.5%), health post (24.4%), and primary health care (69.1%) facilities. The chi-square result showed a statistically significant relationship between the PHC facilities (38.7%, *Χ*² = 7.182, n = 196) and the lack of an established NHMIS mechanism for recording and transmitting service statistics or registers.

When binary logistic regression was used to analyze the data regarding the adequacy of health professionals to provide BMPHS using BHCPF, comprehensive health centers were four times more likely to have inadequate health professionals compared to primary health care facilities and health post facilities (Odds Ratio (AOR) = 4.440, 95% CI = 1.58–12.450; *p-value 0.005*). When comparing respondents with a diploma and those with a degree, those with a diploma were four times more likely to have the NHMIS in their facilities (adjusted odds ratio (AOR) = 4.955, 95% confidence interval (CI) = 1.120–21.036; P-value 0.035). When comparing primary health care facilities to comprehensive health centers and health posts, PHC facilities were two times more likely to have the NHMIS (odds ratio (AOR) = 2.549, 95% CI = 1.167–5.566; *p-value 0. 019)*.

### Challenges associated with BHCPF

**Fig. 1:**
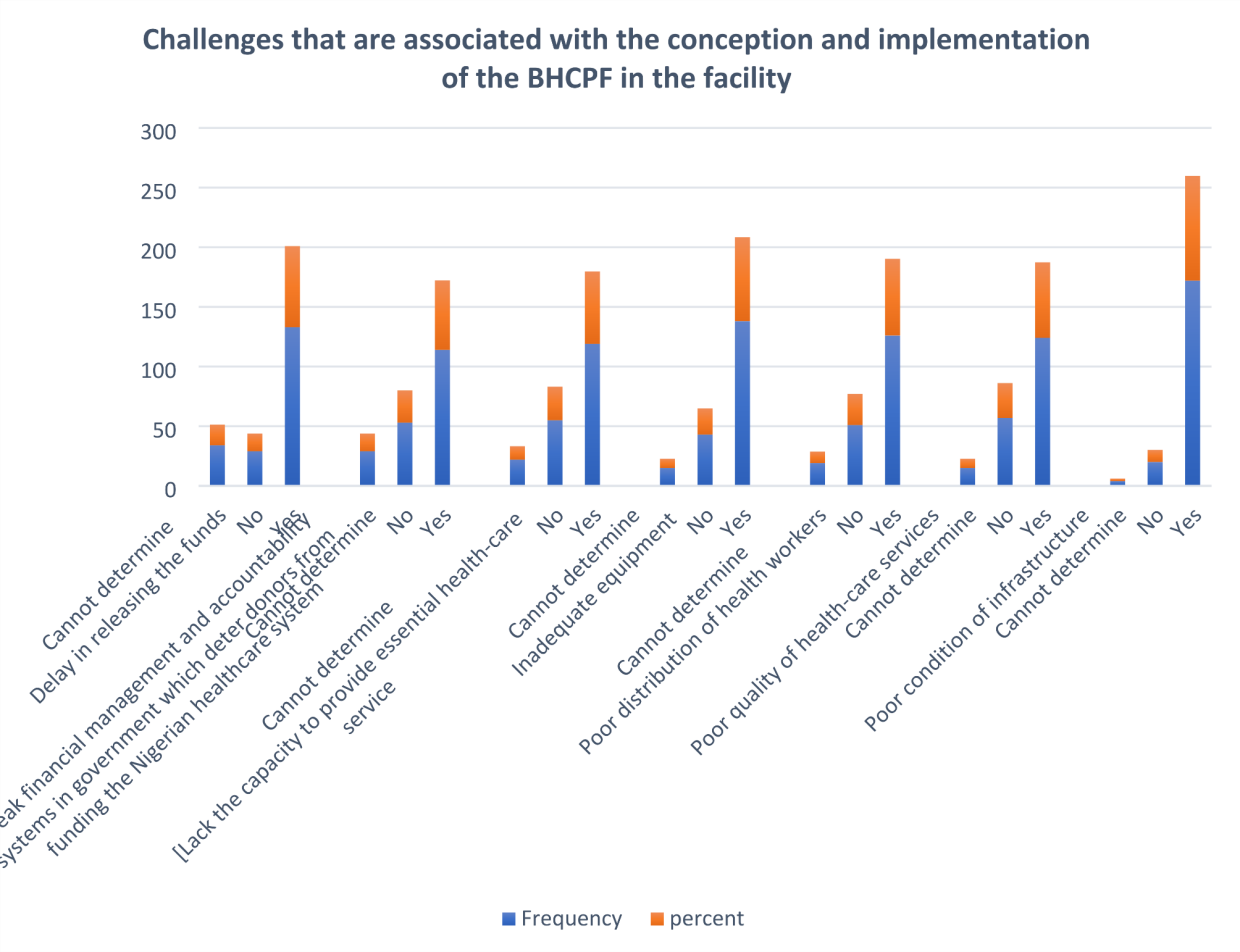
Challenges associated with the conception and implementation of the BHCPF in the facilities

The study revealed challenges associated with the conception and implementation of the BHCPF. The challenges of the BHCPF were funding delays (67.9%), poor financial management, and accountability mechanisms (58.2%). Facilities have a low capacity to offer essential healthcare services (60.7%), challenges with medical equipment in facilities (70.4%), poor staffing (65.8%), and poor infrastructure (87.8%). The challenges associated with the implementation of the BHCPF in healthcare facilities are shown in Fig. 1.

## Discussion

This study assessed the ability of the PHCs to utilize BHCPF to improve health care services in the Kano state of Nigeria. Several factors were identified that relate to the benefits and challenges of the PHC’s ability to improve healthcare services in the selected communities. Most of those who participated in the study were community health extension workers (83.2%), the most significant proportion of health professionals at PHC facilities. The BHCPF guidelines stipulated that each PHC facility should have at least one trained skilled birth attendant, i.e., a midwife or a nurse [5]. According to the WHO technical series on PHC, the workforce should include all professions involved in health promotion and disease prevention and work to address the social determinants of health [12]. Health professionals (93.9%) had a limited level of education, as the diploma was the highest. The World Health Organization stressed professional growth and in-service training in developing and maintaining high-quality PHC services [13]. Through in-service training, healthcare workers can acquire new skills or retrain existing health professionals to deliver effective PHC services in health systems that have not yet built a robust specialized workforce, such as those in rural areas or those with limited resources [13].

Primary health care professionals had awareness (50.5%) and good knowledge (61.7%) of the BHCPF and had received related training, even though some requested additional training (55.6%). Training for the BHCPF should be focused on the specific role of the BHCPF in providing PHC service in rural settings in Nigeria. Primary health care health professionals must have adequate knowledge of the goals of the BHCPF. The majority of healthcare professionals affirmed that they were educated on the importance of making returns, which was included in the training manual. The BPHCPF manual is expected to guide the implementation of activities of the fund. The manual is key in giving information on how to make returns, implement services, and the general organization of the service.

The survey shows that most healthcare workers elucidated that the number of healthcare professionals providing BMPHS in the PHC facilities is inadequate. According to WHO, health professionals are essential for the function of health systems, improving health service coverage, and achieving the right to the highest attainable standard of health [14]. The world health authority also projected an 18 million health professional shortage by 2030, with the majority coming from low- and middle-income countries [14]. The BHCPF project helps periodically evaluate the selected PHC facilities and will be essential in improving healthcare services, solving problems, making informed decisions, and building knowledge. In this study, the majority of healthcare professionals indicated that the BHCPF project allows for periodic evaluation of the PHC facilities, which will aid in determining the quality of the facilities. Healthcare facilities that provide essential services, especially in resource-limited settings, must be periodically monitored to ensure that the minimum acceptable services are provided to community members [15].

Given that health service is challenged in Nigeria, having a system that appropriately coordinates evaluation and monitoring systems, especially in PHC institutions, is cardinal to attaining health service goals. The importance of adequate monitoring was further highlighted when healthcare professionals showed no NHMIS or register for proper record-keeping. The lack of this data management system in PHC facilities reveals a data management gap. Health records must be complete and well-organized to sustain best practices, aid in clear communication between professionals, and improve good medical practice and continuity of care [16]. The BHCPF is a critical component useful in the PHC system by promoting steady, effective monitoring systems. Records maintenance is useful for PHC systems to monitor and determine the progress made in service delivery in poor and remote settings [16].

Healthcare providers identified a lack of exposure to vital information that will make the community members aware of the services of the BMPHS through the BHCPF funding. This emphasizes the gap that needs to be covered through community education on BHCPF. This is because communities are not aware of the primary goal of the BHCPF program. Some studies have shown that it is becoming more widely acknowledged that community participation is essential to making health services more acceptable and sustainable [17]. The promotion of community awareness is an essential component of the strategy to improve access to health care by collaborating with health professionals, and communities, by providing adequate resources, knowledge, and skills that enable the community to make high-quality, educated decisions regarding preventative health measures, diagnosis, treatment, care, and support [18]. The referral system of the PHC facilities was very poor, and some studies have shown that referrals are essential when a patient’s health concerns exceed the scope of primary care. Referral systems are primary health care’s most common and crucial components [19]. Nigeria’s health system lacks a proper link between PHC and higher-level healthcare facilities [20]. In Iran, self-referral was identified as one of the most significant problems with the country’s referral system [21]. Poor referral systems have some unpleasant consequences, especially in service delivery. These poor referral systems are not only peculiar in Nigeria but are also influenced by (a) the distance between a patient’s home and a healthcare facility, (b) the inability to gain access, (c) the cost of transportation, (d) costs of accessing healthcare, such as medicines and laboratory testing, and (e) unjustified payments sought by healthcare providers [19].

A significant number of respondents doubted the project’s continuity because many health-related programs in Nigeria had been unsuccessful. Due to obvious financial, technical, social, and environmental constraints, Nigerians are generally skeptical about the likely sustainability of health service programs [22]. Although the study identified several challenges, the most common were delays in the release of funds, weak financial management, and accountability systems in government, lack of political will, lack of capacity to provide essential healthcare services, insufficient distribution of health workers, the poor state of infrastructure, and lack of requisite personnel/staffing. Inadequate financial resources, poor accountability and governance systems, and insufficient health resources for expanding universal health coverage have been identified as major challenges to delivering quality healthcare services in Africa [23]. Government must develop a political will with the focus of improving on providing universal health coverage for underprivileged communities [22, 23].

This study showed the factors that influence the provision of BHCPF in the primary health care system in Kano state of Nigeria. This study primarily brings the concerns associated with health financing in Nigeria. However, one limitation worth noting is that the authors did not use a scoring scale to assess participants’ knowledge regarding BHCPF. Future research in this area should consider using a standardized scoring scale to assess the knowledge of participants regarding BHCPF. Another limitation of this study was that while the reliability of the test instrument was appropriately assessed and reported on, the researchers did not assess the validity.

## Conclusion

In line with the findings, PHC professionals were knowledgeable about the BHCPF project. The project funding was used to improve the primary health facilities that were selected for the BHCPF program (equipment, drugs, renovation of some structures, etc.). The factors that influenced PHC facilities to promote the implementation of BHCPF included: periodic evaluation of the facilities, availability of functional storage facilities, and improving the standard of care in PHC facilities. There is a need for retraining healthcare workers and creating more community awareness of the BHCPF. The key challenges associated with the BHCPF implementation were inadequate staff to provide BMPHS, poor community awareness of the program, delay in releasing funds, doubting the program’s sustainability, and a poor referral system. Therefore, we suggest an urgent need to increase community awareness and advocacy of the BHCPF program and the strategies for which they might benefit from the funding. It is necessary to design strategies for sustaining the program before the designated period elapses. Further research on the level of community awareness of the BHCPF program should be conducted.

## Data Availability

All data generated or analyzed during this study are included in this published article.

## Abbreviations

BHCPF: Basic Health Care Provision Fund
BMPHS: Basic Minimum Package of Healthcare Services
EMT: Emergency Medical Treatment
NHAct: National Health Act
OOP: Out-of-Pocket Expenditure
PHC: Primary Health Care
SDGs: Sustainable Development Goals
UHC: Universal Health Coverage
UN IGME: United Nations Inter-Agency Group for Child Mortality Estimation
UN: United Nations
UNICEF: United Nations Children’s Fund
WHO: World Health Organization
